# Supplementary material for: A systematic review of the distribution and prevalence of viruses detected in the Peromyscus maniculatus species complex (Rodentia: Cricetidae)
Source: PLoS Pathog. 2025 Jun 10;21(6):e1013125. doi: 10.1371/journal.ppat.1013125 (PMC12201646; doi:10.1371/journal.ppat.1013125)
Supplement: S1 Fig — The sequence label in red is the Sin Nombre reference sequence from NCBI. Sequences highlighted in pink are El Moro Canyon virus (Orthohantavirus carrizalense) collected in 1996 by Rawlings et al. [36]. P. maniculatus sequences highlighted in blue are used for orientation across the S, M, and L segments, since these sequences are all derived from a single study [37]. (PDF) [file ppat.1013125.s002.pdf]

Supplementary Figures

Figure S1 (next page): Phylogeny of the hantavirus S genome segment based on a maximum-likelihood alignment of hantavirus nucleotide sequences collected from *Peromyscus maniculatus*. The sequence label in red is the Sin Nombre reference sequence from NCBI. Sequences highlighted in pink are El Moro Canyon virus (*Orthohantavirus carrizalense*) collected in 1996 by Rawlings et al. (36). *P. maniculatus* sequences highlighted in blue are used for orientation across the S, M, and L segments, since these sequences are all derived from a single study (37).

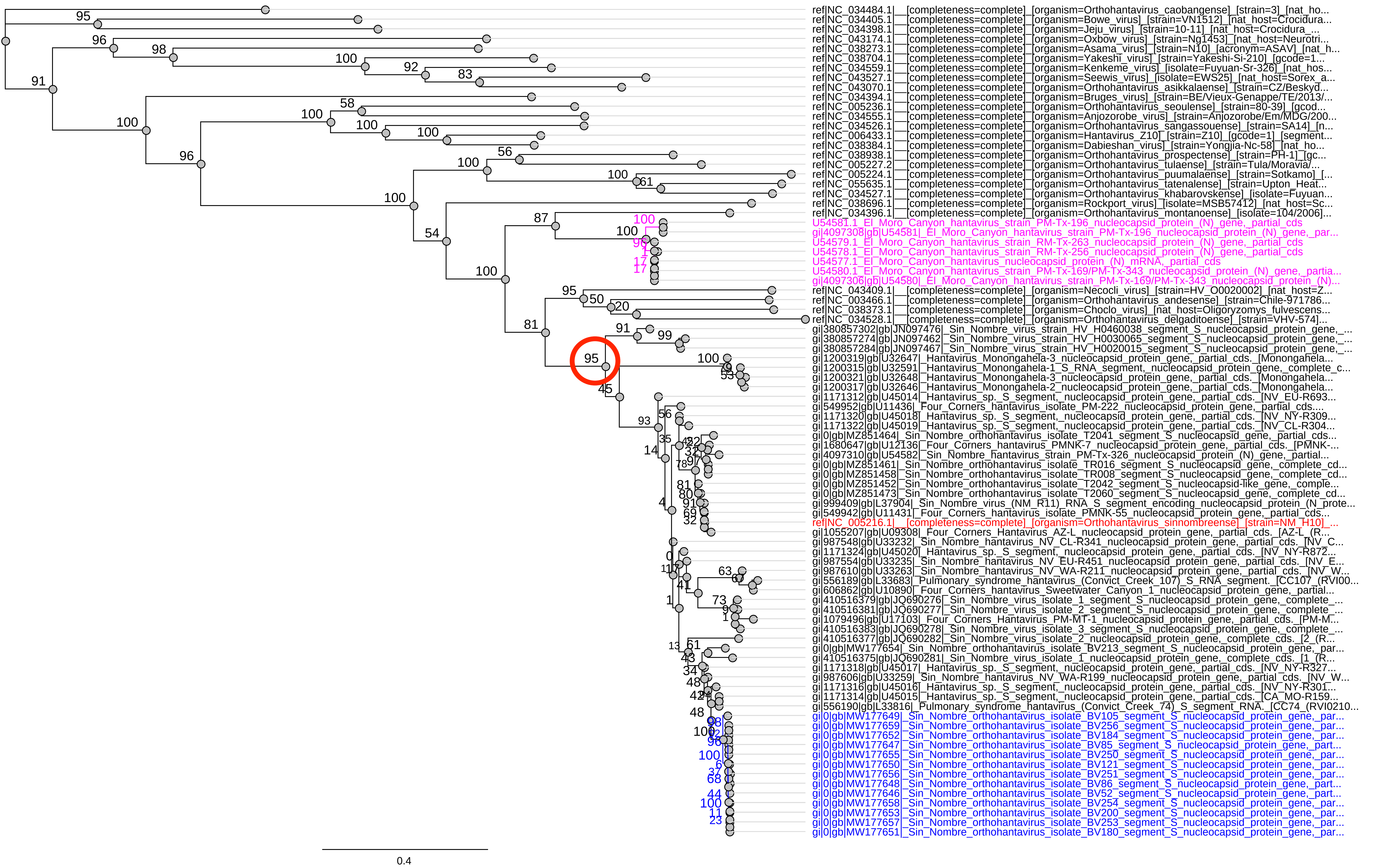

ref|NC_034484.1|__[completeness=complete]_[organism=Orthohantavirus_caobangense]_[strain=3]_[nat_ho...
ref|NC_034405.1|__[completeness=complete]_[organism=Bowe_virus]_[strain=VN1512]_[nat_host=Crocidura...
ref|NC_034398.1|__[completeness=complete]_[organism=Jeju_virus]_[strain=10-11]_[nat_host=Crocidura_...
ref|NC_043174.1|__[completeness=complete]_[organism=Oxbow_virus]_[strain=Ng1453]_[nat_host=Neurotri...
ref|NC_038273.1|__[completeness=complete]_[organism=Asama_virus]_[strain=N10]_[acronym=ASAV]_[nat_h...
ref|NC_038704.1|__[completeness=complete]_[organism=Yakeshi_virus]_[strain=Yakeshi-Si-210]_[gcode=1...
ref|NC_034559.1|__[completeness=complete]_[organism=Kenkeme_virus]_[isolate=Fuyuan-Sr-326]_[nat_hos...
ref|NC_043527.1|__[completeness=complete]_[organism=Seewis_virus]_[isolate=EWS25]_[nat_host=Sorex_a...
ref|NC_043070.1|__[completeness=complete]_[organism=Orthohantavirus_asikkalaense]_[strain=CZ/Beskyd...
ref|NC_034394.1|__[completeness=complete]_[organism=Bruges_virus]_[strain=BE/Vieux-Genappe/TE/2013/...
ref|NC_005236.1|__[completeness=complete]_[organism=Orthohantavirus_seoulense]_[strain=80-39]_[gcod...
ref|NC_034555.1|__[completeness=complete]_[organism=Anjozorobe_virus]_[strain=Anjozorobe/Em/MDG/200...
ref|NC_034526.1|__[completeness=complete]_[organism=Orthohantavirus_sangassouense]_[strain=SA14]_[n...
ref|NC_006433.1|__[completeness=complete]_[organism=Hantavirus_Z10]_[strain=Z10]_[gcode=1]_[segment...
ref|NC_038384.1|__[completeness=complete]_[organism=Dabieshan_virus]_[strain=Yongjia-Nc-58]_[nat_ho...
ref|NC_038938.1|__[completeness=complete]_[organism=Orthohantavirus_prospectense]_[strain=PH-1]_[gc...
ref|NC_005227.2|__[completeness=complete]_[organism=Orthohantavirus_tulaense]_[strain=Tula/Moravia/...
ref|NC_005224.1|__[completeness=complete]_[organism=Orthohantavirus_puumalaense]_[strain=Sotkamo]_[...
ref|NC_055635.1|__[completeness=complete]_[organism=Orthohantavirus_tatenalense]_[strain=Upton_Heat...
ref|NC_034527.1|__[completeness=complete]_[organism=Orthohantavirus_khabarovskense]_[isolate=Fuyuan...
ref|NC_038696.1|__[completeness=complete]_[organism=Rockport_virus]_[isolate=MSB57412]_[nat_host=Sc...
ref|NC_034396.1|__[completeness=complete]_[organism=Orthohantavirus_montanoense]_[isolate=104/2006]...
U54581.1_El_Moro_Canyon_hantavirus_strain_PM-Tx-196_nucleocapsid_protein_(N)_gene,_partial_cds
gi|4097308|gb|U54581|_El_Moro_Canyon_hantavirus_strain_PM-Tx-196_nucleocapsid_protein_(N)_gene,_par...
U54579.1_El_Moro_Canyon_hantavirus_strain_RM-Tx-263_nucleocapsid_protein_(N)_gene,_partial_cds
U54578.1_El_Moro_Canyon_hantavirus_strain_RM-Tx-256_nucleocapsid_protein_(N)_gene,_partial_cds
U54577.1_El_Moro_Canyon_hantavirus_nucleocapsid_protein_(N)_mRNA,_partial_cds
U54580.1_El_Moro_Canyon_hantavirus_strain_PM-Tx-169/PM-Tx-343_nucleocapsid_protein_(N)_gene,_partia...
gi|4097306|gb|U54580|_El_Moro_Canyon_hantavirus_strain_PM-Tx-169/PM-Tx-343_nucleocapsid_protein_(N)...
ref|NC_043409.1|__[completeness=complete]_[organism=Necocli_virus]_[strain=HV_O0020002]_[nat_host=Z...
ref|NC_003466.1|__[completeness=complete]_[organism=Orthohantavirus_andesense]_[strain=Chile-971786...
ref|NC_038373.1|__[completeness=complete]_[organism=Choclo_virus]_[nat_host=Oligoryzomys_fulvescens...
ref|NC_034528.1|__[completeness=complete]_[organism=Orthohantavirus_delgaditoense]_[strain=VHV-574]...
gi|380857302|gb|JN097476|_Sin_Nombre_virus_strain_HV_H0460038_segment_S_nucleocapsid_protein_gene,_...
gi|380857274|gb|JN097462|_Sin_Nombre_virus_strain_HV_H0030065_segment_S_nucleocapsid_protein_gene,_...
gi|380857284|gb|JN097467|_Sin_Nombre_virus_strain_HV_H0020015_segment_S_nucleocapsid_protein_gene,_...
gi|1200319|gb|U32647|_Hantavirus_Monongahela-3_nucleocapsid_protein_gene,_partial_cds._[Monongahela...
gi|1200315|gb|U32591|_Hantavirus_Monongahela-1_S_RNA_segment,_nucleocapsid_protein_gene,_complete_c...
gi|1200321|gb|U32648|_Hantavirus_Monongahela-3_nucleocapsid_protein_gene,_partial_cds._[Monongahela...
gi|1200317|gb|U32646|_Hantavirus_Monongahela-2_nucleocapsid_protein_gene,_partial_cds._[Monongahela...
gi|1171312|gb|U45014|_Hantavirus_sp._S_segment,_nucleocapsid_protein_gene,_partial_cds._[NV_EU-R693...
gi|549952|gb|U11436|_Four_Corners_hantavirus_isolate_PM-222_nucleocapsid_protein_gene,_partial_cds....
gi|1171320|gb|U45018|_Hantavirus_sp._S_segment,_nucleocapsid_protein_gene,_partial_cds._[NV_NY-R309...
gi|1171322|gb|U45019|_Hantavirus_sp._S_segment,_nucleocapsid_protein_gene,_partial_cds._[NV_CL-R304...
gi|0|gb|MZ851464|_Sin_Nombre_orthohantavirus_isolate_T2041_segment_S_nucleocapsid_gene,_partial_cds...
gi|1680647|gb|U12136|_Four_Corners_hantavirus_PMNK-7_nucleocapsid_protein_gene,_partial_cds._[PMNK-...
gi|4097310|gb|U54582|_Sin_Nombre_hantavirus_strain_PM-Tx-326_nucleocapsid_protein_(N)_gene,_partial...
gi|0|gb|MZ851461|_Sin_Nombre_orthohantavirus_isolate_TR016_segment_S_nucleocapsid_gene,_complete_cd...
gi|0|gb|MZ851458|_Sin_Nombre_orthohantavirus_isolate_TR008_segment_S_nucleocapsid_gene,_complete_cd...
gi|0|gb|MZ851452|_Sin_Nombre_orthohantavirus_isolate_T2042_segment_S_nucleocapsid-like_gene,_comple...
gi|0|gb|MZ851473|_Sin_Nombre_orthohantavirus_isolate_T2060_segment_S_nucleocapsid_gene,_complete_cd...
gi|999409|gb|L37904|_Sin_Nombre_virus_(NM_R11)_RNA_S_segment_encoding_nucleocapsid_protein_(N_prote...
gi|549942|gb|U11431|_Four_Corners_hantavirus_isolate_PMNK-55_nucleocapsid_protein_gene,_partial_cds...
ref|NC_005216.1|__[completeness=complete]_[organism=Orthohantavirus_sinnombreense]_[strain=NM_H10]_...
gi|1055207|gb|U09308|_Four_Corners_Hantavirus_AZ-L_nucleocapsid_protein_gene,_partial_cds._[AZ-L_(R...
gi|987548|gb|U33232|_Sin_Nombre_hantavirus_NV_CL-R341_nucleocapsid_protein_gene,_partial_cds._[NV_C...
gi|1171324|gb|U45020|_Hantavirus_sp._S_segment,_nucleocapsid_protein_gene,_partial_cds._[NV_NY-R872...
gi|987554|gb|U33235|_Sin_Nombre_hantavirus_NV_EU-R451_nucleocapsid_protein_gene,_partial_cds._[NV_E...
gi|987610|gb|U33263|_Sin_Nombre_hantavirus_NV_WA-R211_nucleocapsid_protein_gene,_partial_cds._[NV_W...
gi|556189|gb|L33683|_Pulmonary_syndrome_hantavirus_(Convict_Creek_107)_S_RNA_segment._[CC107_(RVI00...
gi|606862|gb|U10890|_Four_Corners_hantavirus_Sweetwater_Canyon_1_nucleocapsid_protein_gene,_partial...
gi|410516379|gb|JQ690276|_Sin_Nombre_virus_isolate_1_segment_S_nucleocapsid_protein_gene,_complete_...
gi|410516381|gb|JQ690277|_Sin_Nombre_virus_isolate_2_segment_S_nucleocapsid_protein_gene,_complete_...
gi|1079496|gb|U17103|_Four_Corners_Hantavirus_PM-MT-1_nucleocapsid_protein_gene,_partial_cds._[PM-M...
gi|410516383|gb|JQ690278|_Sin_Nombre_virus_isolate_3_segment_S_nucleocapsid_protein_gene,_complete_...
gi|410516377|gb|JQ690282|_Sin_Nombre_virus_isolate_2_nucleocapsid_protein_gene,_complete_cds._[2_(R...
gi|0|gb|MW177654|_Sin_Nombre_orthohantavirus_isolate_BV213_segment_S_nucleocapsid_protein_gene,_par...
gi|410516375|gb|JQ690281|_Sin_Nombre_virus_isolate_1_nucleocapsid_protein_gene,_complete_cds._[1_(R...
gi|1171318|gb|U45017|_Hantavirus_sp._S_segment,_nucleocapsid_protein_gene,_partial_cds._[NV_NY-R327...
gi|987606|gb|U33259|_Sin_Nombre_hantavirus_NV_WA-R199_nucleocapsid_protein_gene,_partial_cds._[NV_W...
gi|1171316|gb|U45016|_Hantavirus_sp._S_segment,_nucleocapsid_protein_gene,_partial_cds._[NV_NY-R301...
gi|1171314|gb|U45015|_Hantavirus_sp._S_segment,_nucleocapsid_protein_gene,_partial_cds._[CA_MO-R159...
gi|556190|gb|L33816|_Pulmonary_syndrome_hantavirus_(Convict_Creek_74)_S_segment_RNA._[CC74_(RVI0210...
gi|0|gb|MW177649|_Sin_Nombre_orthohantavirus_isolate_BV105_segment_S_nucleocapsid_protein_gene,_par...
gi|0|gb|MW177659|_Sin_Nombre_orthohantavirus_isolate_BV256_segment_S_nucleocapsid_protein_gene,_par...
gi|0|gb|MW177652|_Sin_Nombre_orthohantavirus_isolate_BV184_segment_S_nucleocapsid_protein_gene,_par...
gi|0|gb|MW177647|_Sin_Nombre_orthohantavirus_isolate_BV85_segment_S_nucleocapsid_protein_gene,_part...
gi|0|gb|MW177655|_Sin_Nombre_orthohantavirus_isolate_BV250_segment_S_nucleocapsid_protein_gene,_par...
gi|0|gb|MW177650|_Sin_Nombre_orthohantavirus_isolate_BV121_segment_S_nucleocapsid_protein_gene,_par...
gi|0|gb|MW177656|_Sin_Nombre_orthohantavirus_isolate_BV251_segment_S_nucleocapsid_protein_gene,_par...
gi|0|gb|MW177648|_Sin_Nombre_orthohantavirus_isolate_BV86_segment_S_nucleocapsid_protein_gene,_part...
gi|0|gb|MW177646|_Sin_Nombre_orthohantavirus_isolate_BV52_segment_S_nucleocapsid_protein_gene,_part...
gi|0|gb|MW177658|_Sin_Nombre_orthohantavirus_isolate_BV254_segment_S_nucleocapsid_protein_gene,_par...
gi|0|gb|MW177653|_Sin_Nombre_orthohantavirus_isolate_BV200_segment_S_nucleocapsid_protein_gene,_par...
gi|0|gb|MW177657|_Sin_Nombre_orthohantavirus_isolate_BV253_segment_S_nucleocapsid_protein_gene,_par...
gi|0|gb|MW177651|_Sin_Nombre_orthohantavirus_isolate_BV180_segment_S_nucleocapsid_protein_gene,_par...
0.4
