# Supplementary material for: A systematic review of the distribution and prevalence of viruses detected in the Peromyscus maniculatus species complex (Rodentia: Cricetidae)
Source: PLoS Pathog. 2025 Jun 10;21(6):e1013125. doi: 10.1371/journal.ppat.1013125 (PMC12201646; doi:10.1371/journal.ppat.1013125)
Supplement: S2 Fig — The sequence label in red is the Sin Nombre reference sequence from NCBI. P. maniculatus sequences highlighted in blue are used for orientation across the S, M, and L segments, since these sequences are all derived from a single study [37]. (PDF) [file ppat.1013125.s003.pdf]

Figure S2 (next page): Phylogeny of the hantavirus M genome segment based on a maximum-likelihood alignment of hantavirus nucleotide sequences collected from *Peromyscus maniculatus*. The sequence label in red is the Sin Nombre reference sequence from NCBI. *P. maniculatus* sequences highlighted in blue are used for orientation across the S, M, and L segments, since these sequences are all derived from a single study (37).

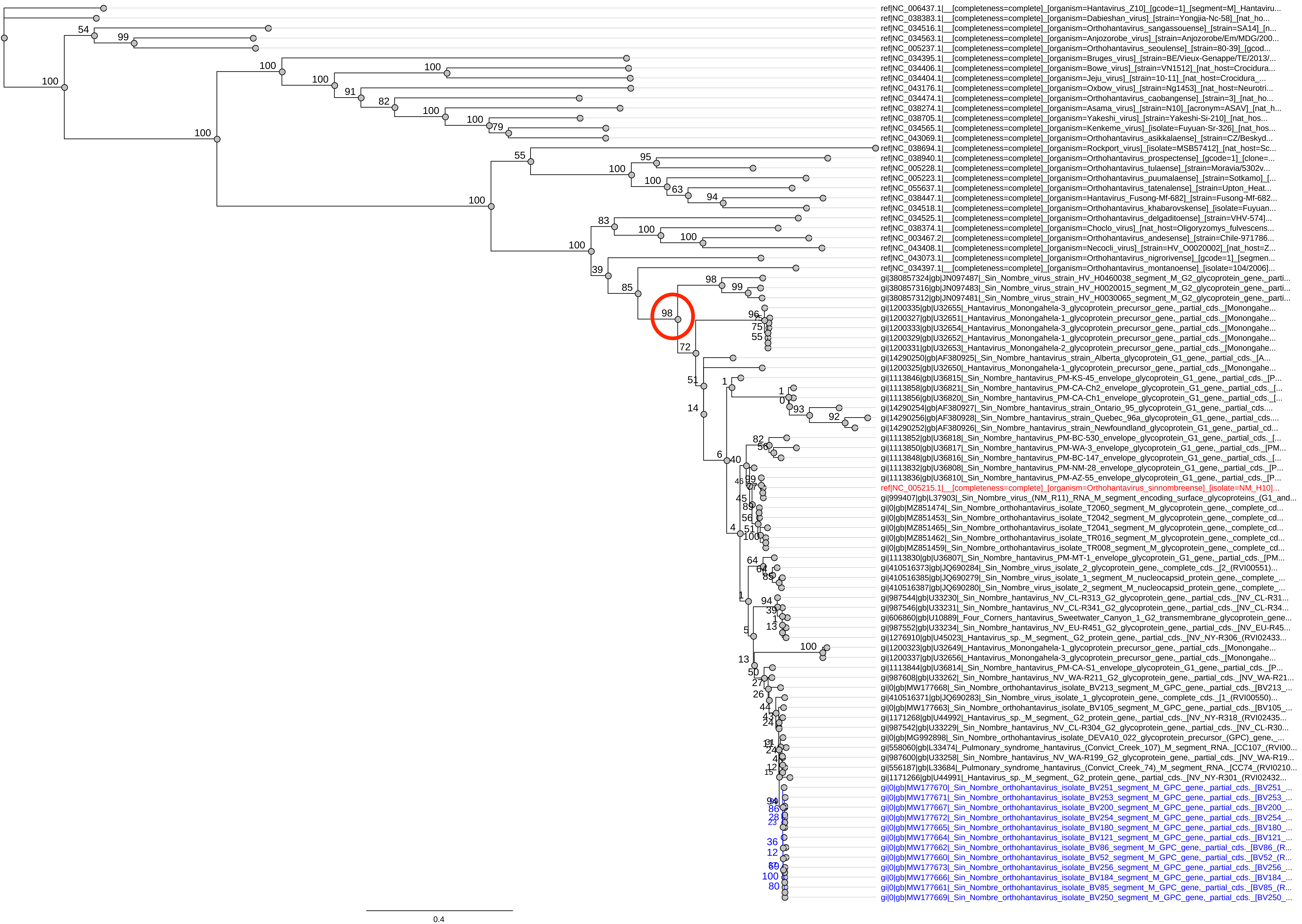
ref|NC_006437.1|__[completeness=complete]_[organism=Hantavirus_Z10]_[gcode=1]_[segment=M]_Hantaviru...
ref|NC_038383.1|__[completeness=complete]_[organism=Dabieshan_virus]_[strain=Yongjia-Nc-58]_[nat_ho...
ref|NC_034516.1|__[completeness=complete]_[organism=Orthohantavirus_sangassouense]_[strain=SA14]_[n...
ref|NC_034563.1|__[completeness=complete]_[organism=Anjozorobe_virus]_[strain=Anjozorobe/Em/MDG/200...
ref|NC_005237.1|__[completeness=complete]_[organism=Orthohantavirus_seoulense]_[strain=80-39]_[gcod...
ref|NC_034395.1|__[completeness=complete]_[organism=Bruges_virus]_[strain=BE/Vieux-Genappe/TE/2013/...
ref|NC_034406.1|__[completeness=complete]_[organism=Bowe_virus]_[strain=VN1512]_[nat_host=Crocidura...
ref|NC_034404.1|__[completeness=complete]_[organism=Jeju_virus]_[strain=10-11]_[nat_host=Crocidura_...
ref|NC_043176.1|__[completeness=complete]_[organism=Oxbow_virus]_[strain=Ng1453]_[nat_host=Neurotri...
ref|NC_034474.1|__[completeness=complete]_[organism=Orthohantavirus_caobangense]_[strain=3]_[nat_ho...
ref|NC_038274.1|__[completeness=complete]_[organism=Asama_virus]_[strain=N10]_[acronym=ASAV]_[nat_h...
ref|NC_038705.1|__[completeness=complete]_[organism=Yakeshi_virus]_[strain=Yakeshi-Si-210]_[nat_hos...
ref|NC_034565.1|__[completeness=complete]_[organism=Kenkeme_virus]_[isolate=Fuyuan-Sr-326]_[nat_hos...
ref|NC_043069.1|__[completeness=complete]_[organism=Orthohantavirus_asikkalaense]_[strain=CZ/Beskyd...
ref|NC_038694.1|__[completeness=complete]_[organism=Rockport_virus]_[isolate=MSB57412]_[nat_host=Sc...
ref|NC_038940.1|__[completeness=complete]_[organism=Orthohantavirus_prospectense]_[gcode=1]_[clone=...
ref|NC_005228.1|__[completeness=complete]_[organism=Orthohantavirus_tulaense]_[strain=Moravia/5302v...
ref|NC_005223.1|__[completeness=complete]_[organism=Orthohantavirus_puumalaense]_[strain=Sotkamo]_[...
ref|NC_055637.1|__[completeness=complete]_[organism=Orthohantavirus_tatenalense]_[strain=Upton_Heat...
ref|NC_038447.1|__[completeness=complete]_[organism=Hantavirus_Fusong-Mf-682]_[strain=Fusong-Mf-682...
ref|NC_034518.1|__[completeness=complete]_[organism=Orthohantavirus_khabarovskense]_[isolate=Fuyuan...
ref|NC_034525.1|__[completeness=complete]_[organism=Orthohantavirus_delgaditoense]_[strain=VHV-574]...
ref|NC_038374.1|__[completeness=complete]_[organism=Choclo_virus]_[nat_host=Oligoryzomys_fulvescens...
ref|NC_003467.2|__[completeness=complete]_[organism=Orthohantavirus_andesense]_[strain=Chile-971786...
ref|NC_043408.1|__[completeness=complete]_[organism=Necocli_virus]_[strain=HV_O0020002]_[nat_host=Z...
ref|NC_043073.1|__[completeness=complete]_[organism=Orthohantavirus_nigrorivense]_[gcode=1]_[segmen...
ref|NC_034397.1|__[completeness=complete]_[organism=Orthohantavirus_montanoense]_[isolate=104/2006]...
gi|380857324|gb|JN097487|_Sin_Nombre_virus_strain_HV_H0460038_segment_M_G2_glycoprotein_gene,_parti...
gi|380857316|gb|JN097483|_Sin_Nombre_virus_strain_HV_H0020015_segment_M_G2_glycoprotein_gene,_parti...
gi|380857312|gb|JN097481|_Sin_Nombre_virus_strain_HV_H0030065_segment_M_G2_glycoprotein_gene,_parti...
gi|1200335|gb|U32655|_Hantavirus_Monongahela-3_glycoprotein_precursor_gene,_partial_cds._[Monongahe...
gi|1200327|gb|U32651|_Hantavirus_Monongahela-1_glycoprotein_precursor_gene,_partial_cds._[Monongahe...
gi|1200333|gb|U32654|_Hantavirus_Monongahela-3_glycoprotein_precursor_gene,_partial_cds._[Monongahe...
gi|1200329|gb|U32652|_Hantavirus_Monongahela-1_glycoprotein_precursor_gene,_partial_cds._[Monongahe...
gi|1200331|gb|U32653|_Hantavirus_Monongahela-2_glycoprotein_precursor_gene,_partial_cds._[Monongahe...
gi|14290250|gb|AF380925|_Sin_Nombre_hantavirus_strain_Alberta_glycoprotein_G1_gene,_partial_cds._[A...
gi|1200325|gb|U32650|_Hantavirus_Monongahela-1_glycoprotein_precursor_gene,_partial_cds._[Monongahe...
gi|1113846|gb|U36815|_Sin_Nombre_hantavirus_PM-KS-45_envelope_glycoprotein_G1_gene,_partial_cds._[P...
gi|1113858|gb|U36821|_Sin_Nombre_hantavirus_PM-CA-Ch2_envelope_glycoprotein_G1_gene,_partial_cds._[...
gi|1113856|gb|U36820|_Sin_Nombre_hantavirus_PM-CA-Ch1_envelope_glycoprotein_G1_gene,_partial_cds._[...
gi|14290254|gb|AF380927|_Sin_Nombre_hantavirus_strain_Ontario_95_glycoprotein_G1_gene,_partial_cds....
gi|14290256|gb|AF380928|_Sin_Nombre_hantavirus_strain_Quebec_96a_glycoprotein_G1_gene,_partial_cds....
gi|14290252|gb|AF380926|_Sin_Nombre_hantavirus_strain_Newfoundland_glycoprotein_G1_gene,_partial_cd...
gi|1113852|gb|U36818|_Sin_Nombre_hantavirus_PM-BC-530_envelope_glycoprotein_G1_gene,_partial_cds._[...
gi|1113850|gb|U36817|_Sin_Nombre_hantavirus_PM-WA-3_envelope_glycoprotein_G1_gene,_partial_cds._[PM...
gi|1113848|gb|U36816|_Sin_Nombre_hantavirus_PM-BC-147_envelope_glycoprotein_G1_gene,_partial_cds._[...
gi|1113832|gb|U36808|_Sin_Nombre_hantavirus_PM-NM-28_envelope_glycoprotein_G1_gene,_partial_cds._[P...
gi|1113836|gb|U36810|_Sin_Nombre_hantavirus_PM-AZ-55_envelope_glycoprotein_G1_gene,_partial_cds._[P...
ref|NC_005215.1|__[completeness=complete]_[organism=Orthohantavirus_sinnombreense]_[isolate=NM_H10]...
gi|999407|gb|L37903|_Sin_Nombre_virus_(NM_R11)_RNA_M_segment_encoding_surface_glycoproteins_(G1_and...
gi|0|gb|MZ851474|_Sin_Nombre_orthohantavirus_isolate_T2060_segment_M_glycoprotein_gene,_complete_cd...
gi|0|gb|MZ851453|_Sin_Nombre_orthohantavirus_isolate_T2042_segment_M_glycoprotein_gene,_complete_cd...
gi|0|gb|MZ851465|_Sin_Nombre_orthohantavirus_isolate_T2041_segment_M_glycoprotein_gene,_complete_cd...
gi|0|gb|MZ851462|_Sin_Nombre_orthohantavirus_isolate_TR016_segment_M_glycoprotein_gene,_complete_cd...
gi|0|gb|MZ851459|_Sin_Nombre_orthohantavirus_isolate_TR008_segment_M_glycoprotein_gene,_complete_cd...
gi|1113830|gb|U36807|_Sin_Nombre_hantavirus_PM-MT-1_envelope_glycoprotein_G1_gene,_partial_cds._[PM...
gi|410516373|gb|JQ690284|_Sin_Nombre_virus_isolate_2_glycoprotein_gene,_complete_cds._[2_(RVI00551)...
gi|410516385|gb|JQ690279|_Sin_Nombre_virus_isolate_1_segment_M_nucleocapsid_protein_gene,_complete_...
gi|410516387|gb|JQ690280|_Sin_Nombre_virus_isolate_2_segment_M_nucleocapsid_protein_gene,_complete_...
gi|987544|gb|U33230|_Sin_Nombre_hantavirus_NV_CL-R313_G2_glycoprotein_gene,_partial_cds._[NV_CL-R31...
gi|987546|gb|U33231|_Sin_Nombre_hantavirus_NV_CL-R341_G2_glycoprotein_gene,_partial_cds._[NV_CL-R34...
gi|606860|gb|U10889|_Four_Corners_hantavirus_Sweetwater_Canyon_1_G2_transmembrane_glycoprotein_gene...
gi|987552|gb|U33234|_Sin_Nombre_hantavirus_NV_EU-R451_G2_glycoprotein_gene,_partial_cds._[NV_EU-R45...
gi|1276910|gb|U45023|_Hantavirus_sp._M_segment,_G2_protein_gene,_partial_cds._[NV_NY-R306_(RVI02433...
gi|1200323|gb|U32649|_Hantavirus_Monongahela-1_glycoprotein_precursor_gene,_partial_cds._[Monongahe...
gi|1200337|gb|U32656|_Hantavirus_Monongahela-3_glycoprotein_precursor_gene,_partial_cds._[Monongahe...
gi|1113844|gb|U36814|_Sin_Nombre_hantavirus_PM-CA-S1_envelope_glycoprotein_G1_gene,_partial_cds._[P...
gi|987608|gb|U33262|_Sin_Nombre_hantavirus_NV_WA-R211_G2_glycoprotein_gene,_partial_cds._[NV_WA-R21...
gi|0|gb|MW177668|_Sin_Nombre_orthohantavirus_isolate_BV213_segment_M_GPC_gene,_partial_cds._[BV213_...
gi|410516371|gb|JQ690283|_Sin_Nombre_virus_isolate_1_glycoprotein_gene,_complete_cds._[1_(RVI00550)...
gi|0|gb|MW177663|_Sin_Nombre_orthohantavirus_isolate_BV105_segment_M_GPC_gene,_partial_cds._[BV105_...
gi|1171268|gb|U44992|_Hantavirus_sp._M_segment,_G2_protein_gene,_partial_cds._[NV_NY-R318_(RVI02435...
gi|987542|gb|U33229|_Sin_Nombre_hantavirus_NV_CL-R304_G2_glycoprotein_gene,_partial_cds._[NV_CL-R30...
gi|0|gb|MG992898|_Sin_Nombre_orthohantavirus_isolate_DEVA10_022_glycoprotein_precursor_(GPC)_gene,_...
gi|558060|gb|L33474|_Pulmonary_syndrome_hantavirus_(Convict_Creek_107)_M_segment_RNA._[CC107_(RVI00...
gi|987600|gb|U33258|_Sin_Nombre_hantavirus_NV_WA-R199_G2_glycoprotein_gene,_partial_cds._[NV_WA-R19...
gi|556187|gb|L33684|_Pulmonary_syndrome_hantavirus_(Convict_Creek_74)_M_segment_RNA._[CC74_(RVI0210...
gi|1171266|gb|U44991|_Hantavirus_sp._M_segment,_G2_protein_gene,_partial_cds._[NV_NY-R301_(RVI02432...
gi|0|gb|MW177670|_Sin_Nombre_orthohantavirus_isolate_BV251_segment_M_GPC_gene,_partial_cds._[BV251_...
gi|0|gb|MW177671|_Sin_Nombre_orthohantavirus_isolate_BV253_segment_M_GPC_gene,_partial_cds._[BV253_...
gi|0|gb|MW177667|_Sin_Nombre_orthohantavirus_isolate_BV200_segment_M_GPC_gene,_partial_cds._[BV200_...
gi|0|gb|MW177672|_Sin_Nombre_orthohantavirus_isolate_BV254_segment_M_GPC_gene,_partial_cds._[BV254_...
gi|0|gb|MW177665|_Sin_Nombre_orthohantavirus_isolate_BV180_segment_M_GPC_gene,_partial_cds._[BV180_...
gi|0|gb|MW177664|_Sin_Nombre_orthohantavirus_isolate_BV121_segment_M_GPC_gene,_partial_cds._[BV121_...
gi|0|gb|MW177662|_Sin_Nombre_orthohantavirus_isolate_BV86_segment_M_GPC_gene,_partial_cds._[BV86_(R...
gi|0|gb|MW177660|_Sin_Nombre_orthohantavirus_isolate_BV52_segment_M_GPC_gene,_partial_cds._[BV52_(R...
gi|0|gb|MW177673|_Sin_Nombre_orthohantavirus_isolate_BV256_segment_M_GPC_gene,_partial_cds._[BV256_...
gi|0|gb|MW177666|_Sin_Nombre_orthohantavirus_isolate_BV184_segment_M_GPC_gene,_partial_cds._[BV184_...
gi|0|gb|MW177661|_Sin_Nombre_orthohantavirus_isolate_BV85_segment_M_GPC_gene,_partial_cds._[BV85_(R...
gi|0|gb|MW177669|_Sin_Nombre_orthohantavirus_isolate_BV250_segment_M_GPC_gene,_partial_cds._[BV250_...
0.4
