# Supplementary material for: A systematic review of the distribution and prevalence of viruses detected in the Peromyscus maniculatus species complex (Rodentia: Cricetidae)
Source: PLoS Pathog. 2025 Jun 10;21(6):e1013125. doi: 10.1371/journal.ppat.1013125 (PMC12201646; doi:10.1371/journal.ppat.1013125)
Supplement: S3 Fig — The sequence label in red is the Sin Nombre reference sequence from NCBI. P. maniculatus sequences highlighted in blue are used for orientation across the S, M, and L segments, since these sequences are all derived from a single study [37]. (PDF) [file ppat.1013125.s004.pdf]

**Figure S3 (next page):** Phylogeny of the hantavirus L genome segment based on a maximum-likelihood alignment of hantavirus nucleotide sequences collected from *Peromyscus maniculatus*. The sequence label in red is the Sin Nombre reference sequence from NCBI. *P. maniculatus* sequences highlighted in blue are used for orientation across the S, M, and L segments, since these sequences are all derived from a single study (37).

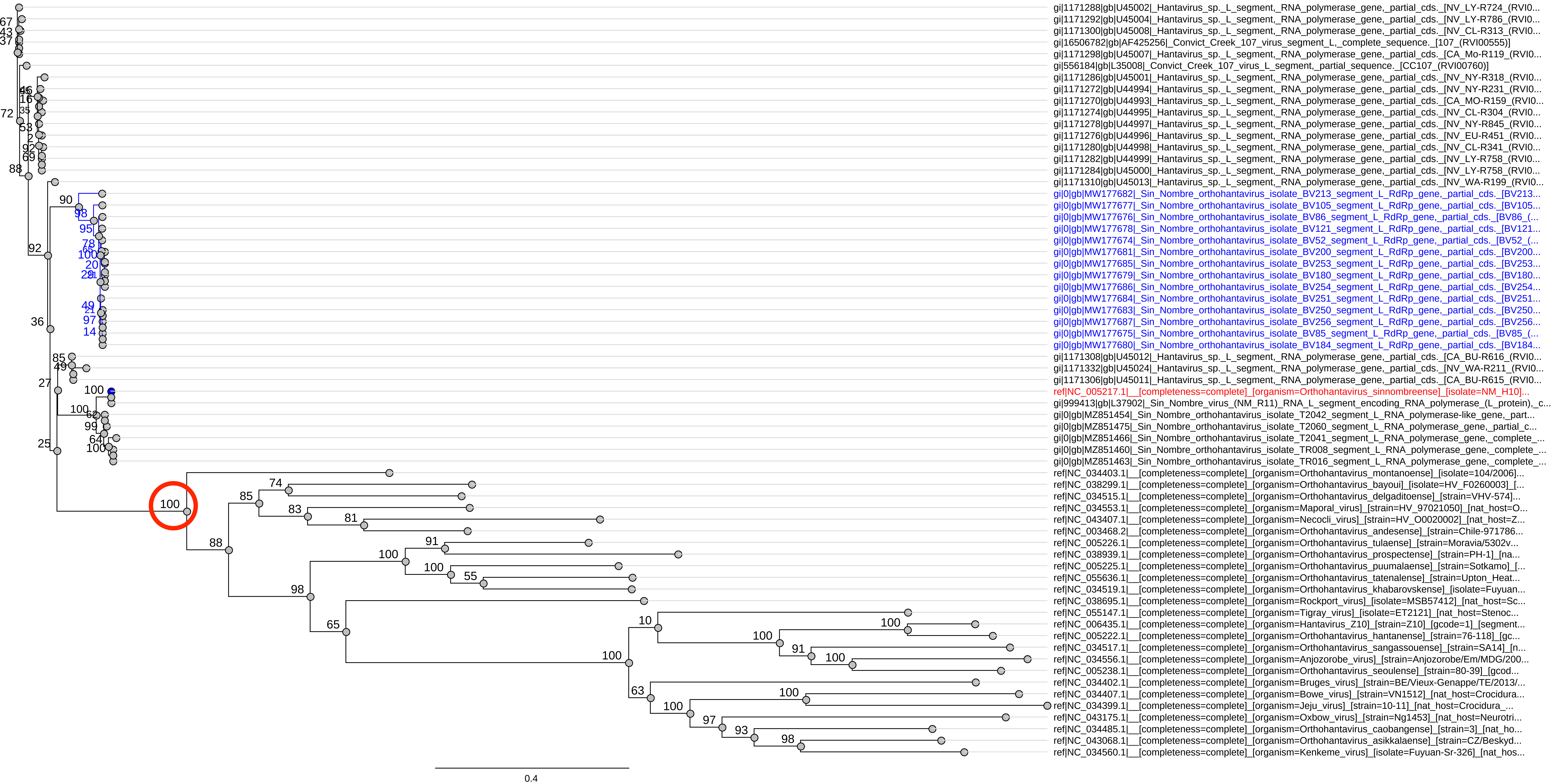

gi|1171288|gb|U45002|_Hantavirus_sp._L_segment,_RNA_polymerase_gene,_partial_cds._[NV_LY-R724_(RVI0...
gi|1171292|gb|U45004|_Hantavirus_sp._L_segment,_RNA_polymerase_gene,_partial_cds._[NV_LY-R786_(RVI0...
gi|1171300|gb|U45008|_Hantavirus_sp._L_segment,_RNA_polymerase_gene,_partial_cds._[NV_CL-R313_(RVI0...
gi|16506782|gb|AF425256|_Convict_Creek_107_virus_segment_L,_complete_sequence._[107_(RVI00555)]
gi|1171298|gb|U45007|_Hantavirus_sp._L_segment,_RNA_polymerase_gene,_partial_cds._[CA_Mo-R119_(RVI0...
gi|556184|gb|L35008|_Convict_Creek_107_virus_L_segment,_partial_sequence._[CC107_(RVI00760)]
gi|1171286|gb|U45001|_Hantavirus_sp._L_segment,_RNA_polymerase_gene,_partial_cds._[NV_NY-R318_(RVI0...
gi|1171272|gb|U44994|_Hantavirus_sp._L_segment,_RNA_polymerase_gene,_partial_cds._[NV_NY-R231_(RVI0...
gi|1171270|gb|U44993|_Hantavirus_sp._L_segment,_RNA_polymerase_gene,_partial_cds._[CA_MO-R159_(RVI0...
gi|1171274|gb|U44995|_Hantavirus_sp._L_segment,_RNA_polymerase_gene,_partial_cds._[NV_CL-R304_(RVI0...
gi|1171278|gb|U44997|_Hantavirus_sp._L_segment,_RNA_polymerase_gene,_partial_cds._[NV_NY-R845_(RVI0...
gi|1171276|gb|U44996|_Hantavirus_sp._L_segment,_RNA_polymerase_gene,_partial_cds._[NV_EU-R451_(RVI0...
gi|1171280|gb|U44998|_Hantavirus_sp._L_segment,_RNA_polymerase_gene,_partial_cds._[NV_CL-R341_(RVI0...
gi|1171282|gb|U44999|_Hantavirus_sp._L_segment,_RNA_polymerase_gene,_partial_cds._[NV_LY-R758_(RVI0...
gi|1171284|gb|U45000|_Hantavirus_sp._L_segment,_RNA_polymerase_gene,_partial_cds._[NV_LY-R758_(RVI0...
gi|1171310|gb|U45013|_Hantavirus_sp._L_segment,_RNA_polymerase_gene,_partial_cds._[NV_WA-R199_(RVI0...
gi|0|gb|MW177682|_Sin_Nombre_orthohantavirus_isolate_BV213_segment_L_RdRp_gene,_partial_cds._[BV213...
gi|0|gb|MW177677|_Sin_Nombre_orthohantavirus_isolate_BV105_segment_L_RdRp_gene,_partial_cds._[BV105...
gi|0|gb|MW177676|_Sin_Nombre_orthohantavirus_isolate_BV86_segment_L_RdRp_gene,_partial_cds._[BV86_(...
gi|0|gb|MW177678|_Sin_Nombre_orthohantavirus_isolate_BV121_segment_L_RdRp_gene,_partial_cds._[BV121...
gi|0|gb|MW177674|_Sin_Nombre_orthohantavirus_isolate_BV52_segment_L_RdRp_gene,_partial_cds._[BV52_(...
gi|0|gb|MW177681|_Sin_Nombre_orthohantavirus_isolate_BV200_segment_L_RdRp_gene,_partial_cds._[BV200...
gi|0|gb|MW177685|_Sin_Nombre_orthohantavirus_isolate_BV253_segment_L_RdRp_gene,_partial_cds._[BV253...
gi|0|gb|MW177679|_Sin_Nombre_orthohantavirus_isolate_BV180_segment_L_RdRp_gene,_partial_cds._[BV180...
gi|0|gb|MW177686|_Sin_Nombre_orthohantavirus_isolate_BV254_segment_L_RdRp_gene,_partial_cds._[BV254...
gi|0|gb|MW177684|_Sin_Nombre_orthohantavirus_isolate_BV251_segment_L_RdRp_gene,_partial_cds._[BV251...
gi|0|gb|MW177683|_Sin_Nombre_orthohantavirus_isolate_BV250_segment_L_RdRp_gene,_partial_cds._[BV250...
gi|0|gb|MW177687|_Sin_Nombre_orthohantavirus_isolate_BV256_segment_L_RdRp_gene,_partial_cds._[BV256...
gi|0|gb|MW177675|_Sin_Nombre_orthohantavirus_isolate_BV85_segment_L_RdRp_gene,_partial_cds._[BV85_(...
gi|0|gb|MW177680|_Sin_Nombre_orthohantavirus_isolate_BV184_segment_L_RdRp_gene,_partial_cds._[BV184...
gi|1171308|gb|U45012|_Hantavirus_sp._L_segment,_RNA_polymerase_gene,_partial_cds._[CA_BU-R616_(RVI0...
gi|1171332|gb|U45024|_Hantavirus_sp._L_segment,_RNA_polymerase_gene,_partial_cds._[NV_WA-R211_(RVI0...
gi|1171306|gb|U45011|_Hantavirus_sp._L_segment,_RNA_polymerase_gene,_partial_cds._[CA_BU-R615_(RVI0...
ref|NC_005217.1|__[completeness=complete]_[organism=Orthohantavirus_sinnombreense]_[isolate=NM_H10]...
gi|999413|gb|L37902|_Sin_Nombre_virus_(NM_R11)_RNA_L_segment_encoding_RNA_polymerase_(L_protein),_c...
gi|0|gb|MZ851454|_Sin_Nombre_orthohantavirus_isolate_T2042_segment_L_RNA_polymerase-like_gene,_part...
gi|0|gb|MZ851475|_Sin_Nombre_orthohantavirus_isolate_T2060_segment_L_RNA_polymerase_gene,_partial_c...
gi|0|gb|MZ851466|_Sin_Nombre_orthohantavirus_isolate_T2041_segment_L_RNA_polymerase_gene,_complete_...
gi|0|gb|MZ851460|_Sin_Nombre_orthohantavirus_isolate_TR008_segment_L_RNA_polymerase_gene,_complete_...
gi|0|gb|MZ851463|_Sin_Nombre_orthohantavirus_isolate_TR016_segment_L_RNA_polymerase_gene,_complete_...
ref|NC_034403.1|__[completeness=complete]_[organism=Orthohantavirus_montanoense]_[isolate=104/2006]...
ref|NC_038299.1|__[completeness=complete]_[organism=Orthohantavirus_bayoui]_[isolate=HV_F0260003]_[...
ref|NC_034515.1|__[completeness=complete]_[organism=Orthohantavirus_delgaditoense]_[strain=VHV-574]...
ref|NC_034553.1|__[completeness=complete]_[organism=Maporal_virus]_[strain=HV_97021050]_[nat_host=O...
ref|NC_043407.1|__[completeness=complete]_[organism=Necocli_virus]_[strain=HV_O0020002]_[nat_host=Z...
ref|NC_003468.2|__[completeness=complete]_[organism=Orthohantavirus_andesense]_[strain=Chile-971786...
ref|NC_005226.1|__[completeness=complete]_[organism=Orthohantavirus_tulaense]_[strain=Moravia/5302v...
ref|NC_038939.1|__[completeness=complete]_[organism=Orthohantavirus_prospectense]_[strain=PH-1]_[na...
ref|NC_005225.1|__[completeness=complete]_[organism=Orthohantavirus_puumalaense]_[strain=Sotkamo]_[...
ref|NC_055636.1|__[completeness=complete]_[organism=Orthohantavirus_tatenalense]_[strain=Upton_Heat...
ref|NC_034519.1|__[completeness=complete]_[organism=Orthohantavirus_khabarovskense]_[isolate=Fuyuan...
ref|NC_038695.1|__[completeness=complete]_[organism=Rockport_virus]_[isolate=MSB57412]_[nat_host=Sc...
ref|NC_055147.1|__[completeness=complete]_[organism=Tigray_virus]_[isolate=ET2121]_[nat_host=Stenoc...
ref|NC_006435.1|__[completeness=complete]_[organism=Hantavirus_Z10]_[strain=Z10]_[gcode=1]_[segment...
ref|NC_005222.1|__[completeness=complete]_[organism=Orthohantavirus_hantanense]_[strain=76-118]_[gc...
ref|NC_034517.1|__[completeness=complete]_[organism=Orthohantavirus_sangassouense]_[strain=SA14]_[n...
ref|NC_034556.1|__[completeness=complete]_[organism=Anjozorobe_virus]_[strain=Anjozorobe/Em/MDG/200...
ref|NC_005238.1|__[completeness=complete]_[organism=Orthohantavirus_seoulense]_[strain=80-39]_[gcod...
ref|NC_034402.1|__[completeness=complete]_[organism=Bruges_virus]_[strain=BE/Vieux-Genappe/TE/2013/...
ref|NC_034407.1|__[completeness=complete]_[organism=Bowe_virus]_[strain=VN1512]_[nat_host=Crocidura...
ref|NC_034399.1|__[completeness=complete]_[organism=Jeju_virus]_[strain=10-11]_[nat_host=Crocidura_...
ref|NC_043175.1|__[completeness=complete]_[organism=Oxbow_virus]_[strain=Ng1453]_[nat_host=Neurotri...
ref|NC_034485.1|__[completeness=complete]_[organism=Orthohantavirus_caobangense]_[strain=3]_[nat_ho...
ref|NC_043068.1|__[completeness=complete]_[organism=Orthohantavirus_asikkalaense]_[strain=CZ/Beskyd...
ref|NC_034560.1|__[completeness=complete]_[organism=Kenkeme_virus]_[isolate=Fuyuan-Sr-326]_[nat_hos...
0.4
