# Supplementary material for: A systematic review of the distribution and prevalence of viruses detected in the Peromyscus maniculatus species complex (Rodentia: Cricetidae)
Source: PLoS Pathog. 2025 Jun 10;21(6):e1013125. doi: 10.1371/journal.ppat.1013125 (PMC12201646; doi:10.1371/journal.ppat.1013125)
Supplement: Attachment — Submitted filename: Response to reviewers-2ndRev - Final.pdf [file ppat.1013125.s013.pdf]

Response to reviewers, 2nd revision

Reviewer 1
—"The authors have addressed the issues from the previous manuscript but have not addressed El Moro Canyon virus that has been detected in deer mice (see PMID 9025697). It is not clear that other hantaviruses might infect deer mice, thus the assertion that seropositive deer mice must be infected with SNV is not justified."

Thank you for following up about this paper, which we had somehow missed in our original sequence alignment. We have added these sequences to our phylogeny and noted them as an important exception in the results and discussion. Indeed, seropositive deer mice could stem from past infection with SNV on El Moro Canyon virus, if not also others. However, since the El Moro Canyon viral sequences were from a 1996 study, and many of the more recent Sin Nombre antibody positives have been validated by PCR tests, the El Moro Canyon positives require additional replications to show they are not a rare exception.

Reviewer #3:

—"The authors have addressed my main concerns in their revised submission. I disagree with the authors responses that the years ecological work conducted on SNV and other hantaviruses are out of scope for this work. I would prefer to see some further discussion on that topic since those studies represent a vast amount of knowledge that is in my opinion highly relevant to this topic."

We have added a paragraph in the discussion that highlights some key results from long-term Sin Nombre monitoring studies and suggest that the extracted data in Table S3 provides an important stepping stone toward a future modeling study that aggregates across field sites and sampling times.
