# Supplementary material for: A systematic review of the distribution and prevalence of viruses detected in the Peromyscus maniculatus species complex (Rodentia: Cricetidae)
Source: PLoS Pathog. 2025 Jun 10;21(6):e1013125. doi: 10.1371/journal.ppat.1013125 (PMC12201646; doi:10.1371/journal.ppat.1013125)
Supplement: Attachment — Submitted filename: Comments Peromyscus systematic review..docx [file ppat.1013125.s010.docx]

The manuscript by Finkbeiner et al. describes a systematic review conducted by the authors, in which they extracted data from all relevant papers looking at natural viral infection of Peromyscus since 2000. Since deer mice are a critical reservoir for SNV and other viruses (not to mention pathogenic bacteria and parasites), this work is a timely description of what studies have been done to date surveying infection of these mice. The authors describe weighted seroprevalence mostly for SNV and describe the strong bias toward sampling in the western United States compared to other regions. They also do a nice job discussing the complexity of how to group together host species and viruses given the recent suggested taxonomic update of the Peromyscus genus, which as they note, means that 92% of described infections need to be reclassified. The manuscript nicely distills information from 46 studies and >60,000 observations into one well-written and well-presented package. I have some comments and questions regarding a few of the authors’ choices.

I am wondering why the authors chose to specifically include “viruses detected in Peromyscus” in the title, rather than simply a scoping review on SNV? As it stands, the arenaviruses and Powassan make up such a minority of the data that this might have made more sense, with just a mention of the other viruses, which is basically how it is already.

In the results section starting with “other viruses were sampled less frequently…” it discusses 1 study with 353 observations for AMAV and WWAV. The mentioned of “arenavirus” tested for in only a single study, while then mentioning multiple studies later on looking at AMAV and WWAV is a bit confusing, even though I know they mean a broader Arenavirus positivity and not one that is species specific. To this end, in the last sentence of this paragraph it also says that there are multiple studies focusing on arenaviruses including WWAV and AMAV, but I think this adds to the confusion without clarifying the difference, because in the preceding paragraph is says there are only 2 studies looking at WWAV and AMAV. I would maybe make this distinction a bit more clear.

Minor comments:

Discussion; sentence starting with “results on Powassan virus,…” it says they are informative as the indicate the presence of these other hantaviruses, though these are not all hantaviruses. And perhaps a brief discussion about Monongahela virus and how it is not its own species as of the most recent taxonomic update might be warranted.

There is a section of the discussion where the authors discuss variability in seroprevalence and how this is likely due to inconsistencies in sampling across locations and timing. It may be worth a brief discussion on what is known about how rates of SNV prevalence change in deer mouse populations depending on season and how the timing of individuals studies might influence the data in this regard. In lieu of not including timing of sampling in individual studies as part of the wider analysis
