# Supplementary material for: A systematic review of the distribution and prevalence of viruses detected in the Peromyscus maniculatus species complex (Rodentia: Cricetidae)
Source: PLoS Pathog. 2025 Jun 10;21(6):e1013125. doi: 10.1371/journal.ppat.1013125 (PMC12201646; doi:10.1371/journal.ppat.1013125)
Supplement: Attachment — Submitted filename: Response to Reviewers - PLoS Pathogens-Dec20.docx [file ppat.1013125.s012.docx]

Editor comments:

**Based on especially Reviewer 3's comments, I'd strongly advise that the authors double-check their searches thoroughly using both "deer mouse", "deer mice", "deermouse" and "deer mice" in addition to "Peromyscus" to ensure both accurate literature citing and scoping… likewise, "deermouse/deermice" (no space) is the correct term for all animals classified in genus _Permomyscus_ per the zoological reference standard (Wilson & Reeder).**

Thanks for raising this issue, and we agree that earlier literature sometimes uses “deer mice” as a common name for *P. maniculatus*. This is technically the common name for the whole genus, though, which includes 82 different species. The current reference standard, the Mammal Diversity Database, uses “Eastern Deermouse” and “North American Deermouse” for *P. maniculatus* – and for other species in the complex: “Western Deermouse” (*P. sonoriensis*), “Gambel's Deermouse” (*P. gambelii*), and “Southern Deermouse” (*P. labecula*).

Nonetheless, the study goal is to include a comprehensive review of viruses detected in the *P. maniculatus* species complex. To this end, we ran supplementary searches in PubMed, PubMed Central, and Scopus. The search queries were constructed to identify any papers that we may have missed because they mentioned only the common names but not *Peromyscus maniculatus*, since any papers that mentioned “*Peromyscus maniculatus”* would have been included in our original search results. The logic of the search query was: (“deermouse” OR “deermice” OR “deer mouse” OR “deer mice”) AND (virus OR viruses OR viral) NOT (“Peromyscus maniculatus”). We searched title and abstract in PubMed, title/abstract and full body of text in PubMedCentral, and title/abstract in Scopus for papers published between 2000 and Aug 2022.

We identified three papers that were not included in our original search results and that have prevalence data for viruses detected in wild *P. maniculatus*. We have extracted these results and added them to our analyses as ad hoc papers. We have added a discussion of this update to our methods.

**As a side note, in response to reviewers: HPS, not HCPS, is the official term for the disease as per the WHO ICD-11, so I would recommend leaving this;**

We have kept the name HPS as recommended.

Reviewer 1

It would have been helpful if the authors had included other viruses hosted by peromyscus rodents, such as Modoc virus and El Moro Canyon virus (which is also found principally in western harvest mice). In the end, the viruses/reservoirs are somewhat "fuzzy" simply because different viruses can be found in multiple species.

We agree that this is a good target for future research, and we are continuing to work on extracting results from all North American rodent species. However, the scope of this study is restricted to the *P. maniculatus* species complex, and expanding it to cover all species in the genus would require analyzing approximately 100 additional studies. Nonetheless, we do report results for all viruses studied in *P. maniculatus*.

The authors should clarify how the serology was done in these studies (e.g., "primary antigen" page 8). There is a huge difference between ELISA with nucleocapsid (highly conserved among New World hantaviruses) and Gn/Gc (more divergent), and assays such as focus reduction tests with cell culture/infectious virus. Antibodies to SNV nucleocapsid are cross reactive with Andes virus nucleocapsid, for example. This can have a profound impact on the analyses and conclusions raised by the authors. This should also be discussed in further detail in the Discussion on page 13 ("virus- specific antibody" tests).

Our thanks to the referee for raising this issue. We have revisited all the papers reporting antibody results and extracted both the antigen target and method citation. These are provided as additional columns in Supplementary Table 2. With a couple exceptions, we found that antibody tests in our dataset used the nucleocapsid protein at their target. One interpretation might therefore be that the studies are in fact only reporting hantavirus detection at the family level. However, we noticed that authors of these studies argue that the results are in fact more specific because only one hantavirus species, Sin Nombre, has been detected in these rodent species.

To investigate this assumption, we downloaded available virus genetic sequences collected from *Peromyscus maniculatus* from the ZOVER database and aligned them to reference sequences from NCBI for other relevant orthohantaviruses. (No sequences were published under the new four names after the maniculatus split.) The results are shown in Supplementary Figure 1. None of the available hantavirus sequences from *P. maniculatus* clearly clustered with a virus species other than Sin Nombre. In summary, current genetic evidence is consistent with the claim that Sin Nombre virus is the only hantavirus species that has been detected in the *P. maniculatus* species complex. This is also supported by a handful of RT-PCR studies that confirmed antibody results. We have therefore preserved how each study reported its virus detection results in our analysis. Nonetheless, one could readily map these results onto the more coarse-grained hantavirus family group using the Supplementary Tables if desired.

We have added paragraphs to the results and methods sections describing this analysis.

Although originally thought to be a pulmonary disease, SNV causes a cardiopulmonary disease. This is why clinical efforts are principally focused on the cardiovascular system. This should be added to the abstract (and why most of us refer to it has HCPS).

Also in the abstract, do not capitalize "hantavirus (cardio)pulmonary syndrome" because it is not a proper noun. Again on page 5.

Per the editor’s instruction, we have kept hantavirus pulmonary syndrome as the disease name but added a note of the alternative name HCPS. We have removed the capitalization.

Author Summary. What is meant by "locked"?

We have revised the sentence to clarify that it means information that is published in scientific articles in a form that is not usable as open data for computational research.

if referring to the families, then use Hantaviridae and Arenaviridae and italicize.

We have updated these names throughout the text and supplements.

I also suggest the authors add "deer mouse" to the keyword list; otherwise, the risk is the paper will not be found by those who search for "deer mouse."

Please see response to editor above.

Change "mortality rate" to "case fatality rate." They are different terms.

Thanks for pointing this out, and we have made the edit.

Figure 3 is out of order. The figures should be in the order they are referenced in the text. Figure 3 should be the last figure.

Thanks for catching this mistake, and we have corrected the ordering.

Reviewer 2

I am wondering why the authors chose to specifically include “viruses detected in Peromyscus” in the title, rather than simply a scoping review on SNV? As it stands, the arenaviruses and Powassan make up such a minority of the data that this might have made more sense, with just a mention of the other viruses, which is basically how it is already.

Thanks for raising this point. We agree that SNV dominates the dataset, but we believe the title most accurately reflects the way that the systematic review was constructed based on the PRISMA standards. The title also reflects the comprehensive review and taxonomic update we provide for the maniculatus complex.

In the results section starting with “other viruses were sampled less frequently...” it discusses 1 study with 353 observations for AMAV and WWAV. The mentioned of “arenavirus” tested for in only a single study, while then mentioning multiple studies later on looking at AMAV and WWAV is a bit confusing, even though I know they mean a broader Arenavirus positivity and not one that is species specific. To this end, in the last sentence of this paragraph it also says that there are multiple studies focusing on arenaviruses including WWAV and AMAV, but I think this adds to the confusion without clarifying the difference, because in the preceding paragraph is says there are only 2 studies looking at WWAV and AMAV. I would maybe make this distinction a bit more clear.

Thanks for pointing this out, and we have streamlined the text to remove this confusion.

Discussion; sentence starting with “results on Powassan virus,...” it says they are informative as the indicate the presence of these other hantaviruses, though these are not all hantaviruses. And perhaps a brief discussion about Monongahela virus and how it is not its own species as of the most recent taxonomic update might be warranted.

Thanks, we have revised the text to remove this confusion and added the update on Monongahela.

There is a section of the discussion where the authors discuss variability in seroprevalence and how this is likely due to inconsistencies in sampling across locations and timing. It may be worth a brief discussion on what is known about how rates of SNV prevalence change in deer mouse populations depending on season and how the timing of individuals studies might influence the data in this regard. In lieu of not including timing of sampling in individual studies as part of the wider analysis.

It is outside the scope of this paper to synthesize results about hantavirus risk indicators, but we have added citations to studies in our dataset that analyzed the effects of factors such as demographics, biotic interactions, and abiotic variables such as temperature or rainfall.

Reviewer 3

**Several references are missing which may suggest flaws in the search terms. For example, Goodfellow et al. 2021 (PMID 34549977) appears to be absent from the listed references, yet it describes similar concerns with the current knowledge of hantavirus / rodent reservoir theories. In my brief searches, this is the third paper that comes up if one searches for "Sin Nombre virus AND deer mice" .**

In fact Goodfellow et al. 2021 was included in our original submission (see Supplementary Table 2). We also added three further studies based on further searching for deer mice/deer mouse.

As stated above the description of virology in this paper needs to be improved. The basic description of virus detection methods lead to confusion and are potentially misleading. Hantaviruses are known to be serologically cross reactive and most studies conducted to date relay on basic ELSIA methodologies to determine the prevalence of hantaviruses in rodents. To determine it is SNV with certainty requires molecular techniques and often sequence analysis, which was frequently not done in earlier studies. Although serotyping ELISAs have been described, they are not commonly used therefore statements like "...studies are focused on SNV antibodies.." misleading. The studies are focused on antibodies reactive to hantaviruses.

Please see our response to the editor on this point above. We have also updated the relevant text.

Authors include Eastern deermouse and North American deermouse in search terms but never simply deer mouse. I get zero results for North American deermouse but get 3,236 hits for deer mouse on Pubmed. Perhaps deer mouse should be included in the search criteria? Is it deermouse or deer mouse? I more commonly see it as deer mouse, yet the search terms used list "deermouse"

Please see our response to the editor on this point above.

In the introduction the authors should differentiate between studies demonstrating susceptibility (which I assume is the term the authors use for experimental infections) versus known to harbor which appears to be based on ecological studies. For example, they cite a study by "Griffin" in 2021 (which appears to be an incomplete reference) suggesting deer mice are susceptible to SARS-Cov-2 but as far as this reviewer is aware deer mice have not been implicated as natural reservoirs for this virus.

We have revised the language to say that there is some evidence suggesting P. maniculatus may potentially be susceptible to infection by these viruses.

**Further, there is no mention of long-term ecology / prevalence studies conducted across the USA post-1993 attempting to define indicators for predicting hantavirus spillover events.**

It is outside the scope of this paper to synthesize results about hantavirus risk indicators, but we have added citations to studies in our dataset that analyzed the effects of factors such as demographics, biotic interactions, and abiotic variables such as temperature or rainfall.

Further, the statement "Results on Powassan virus, Whitewater Arroyo Virus, Amapari virus and Monongahela virus are informative in that they indicate the presence of these other hantaviruses within the P. maniculatus complex" is incorrect. Most of the viruses listed are not hantaviruses. This statement further highlights the incomplete or inaccurate knowledge of viruses presented in this manuscript.

Thanks for pointing this out, and we have corrected this text.
